# Supplementary material for: Overexpression of Maize ZmC1 and ZmR Transcription Factors in Wheat Regulates Anthocyanin Biosynthesis in a Tissue-Specific Manner
Source: Int J Mol Sci. 2019 Nov 19;20(22):5806. doi: 10.3390/ijms20225806 (PMC6887777; doi:10.3390/ijms20225806)
Supplement: Supplementary file 1 [file ijms-20-05806-s001.pdf]

Supplementary Materials:

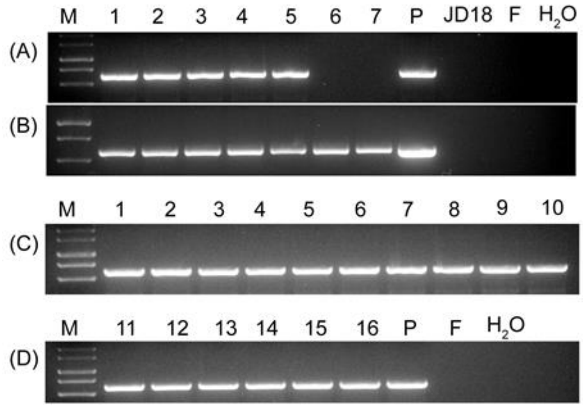

Figure S1. Detection of transgenic wheat plants with *ZmC1* and *ZmR* genes by PCR. **A**: PCR amplification for *ZmC1*; **B**: PCR amplification for *ZmR*; 1-5: transgenic plants transformed with expression vector pWMB202; 6-7: transgenic plants transformed with expression vector pWMB197; P: positive control of vector pWMB202; JD18 and F: negative control of wild type Jingdong18 and Fielder, respectively; H_2O: empty control of sterile water. **C** and **D**: PCR amplification for *ZmC1*; 1-16: transgenic plants transformed with expression vector pWMB196; P: positive control of vector pWMB196; F: negative control of wild type Fielder; H_2O: empty control of sterile water.

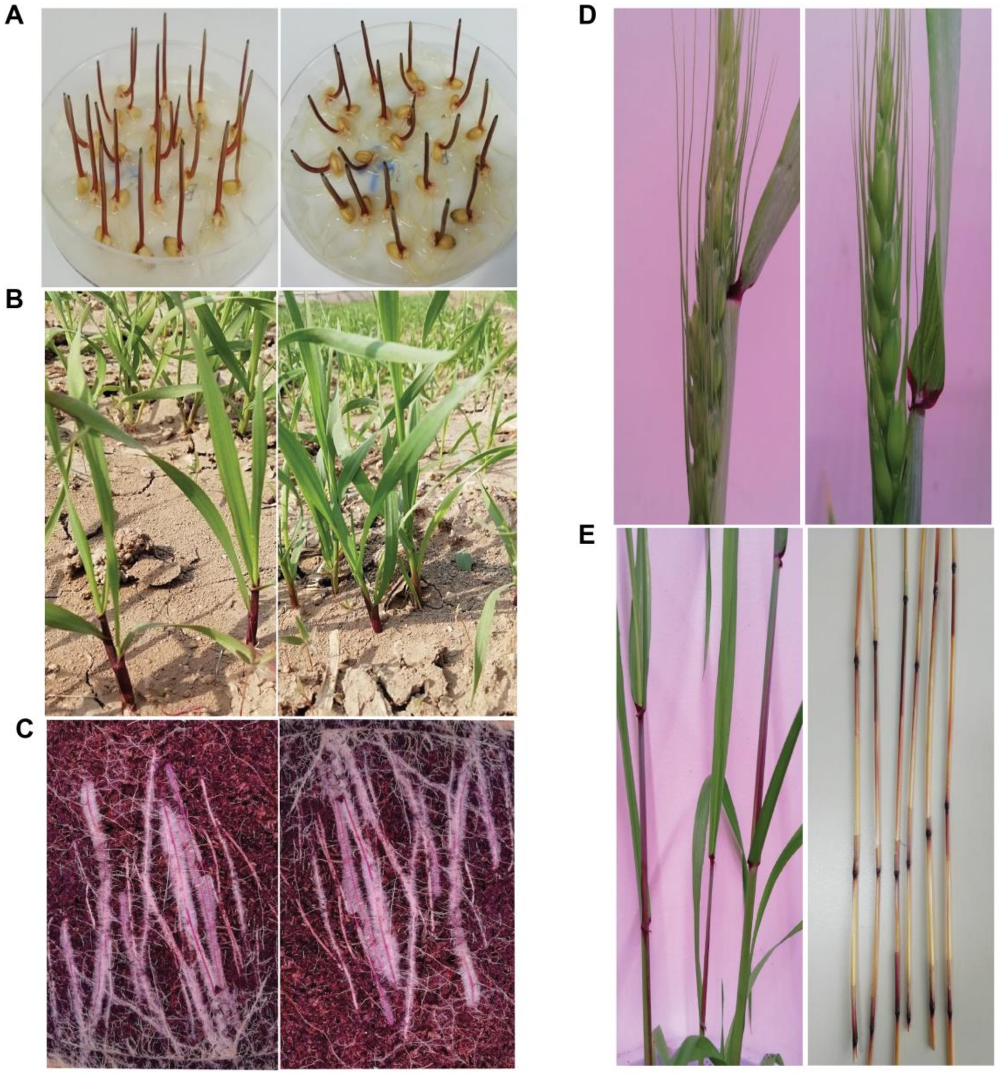

Figure S2. Anthocyanin production in the vegetative tissues of transgenic line AL-31. **A**: coleoptiles; **B**: seedlings; **C**: roots; **D**: auricles; **E**: stems.

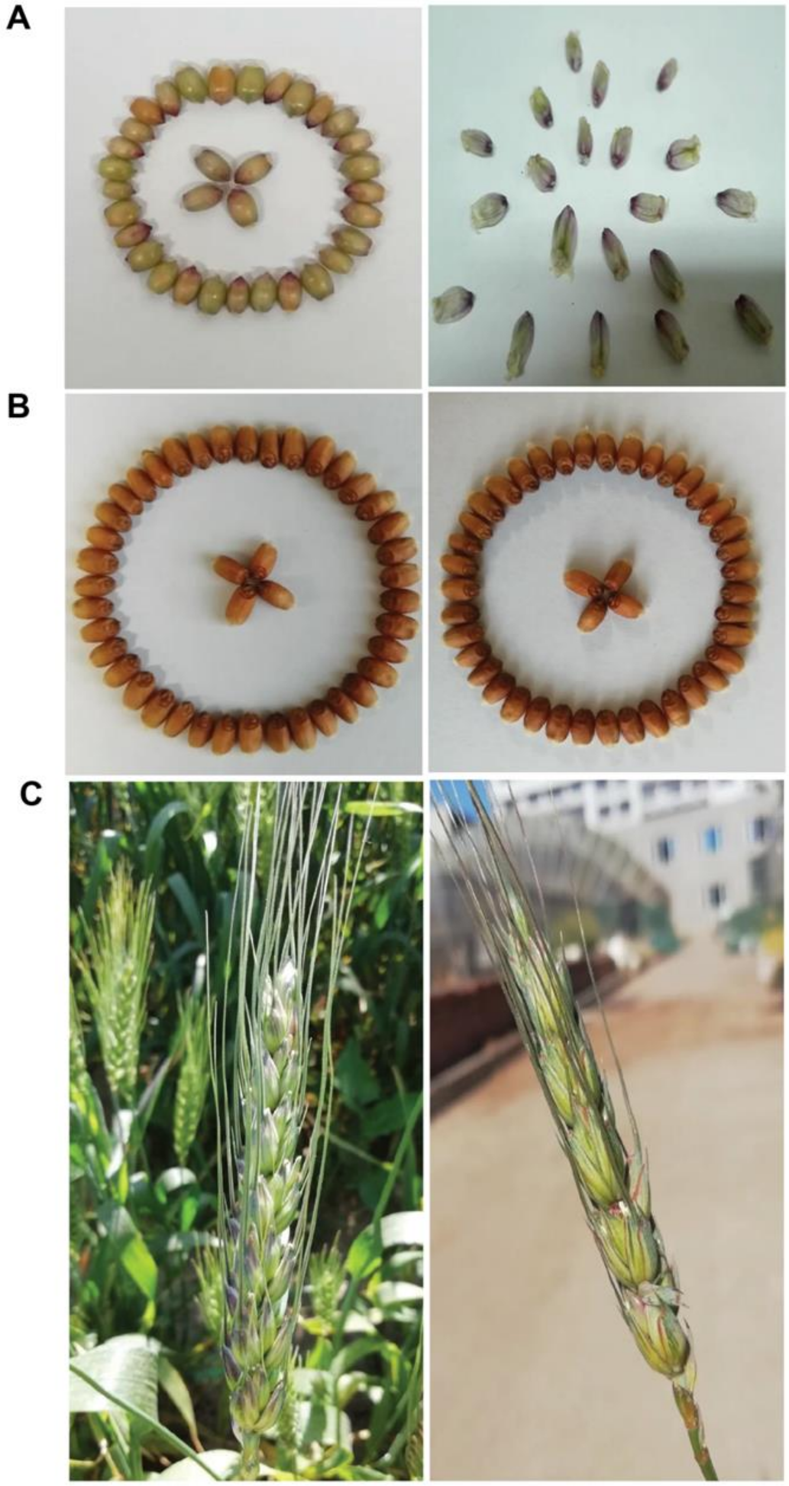

Figure S3. Anthocyanin production in the reproductive tissues of transgenic line AL-41. **A**: immature seeds; **B**: mature seeds; **C**: spikes.

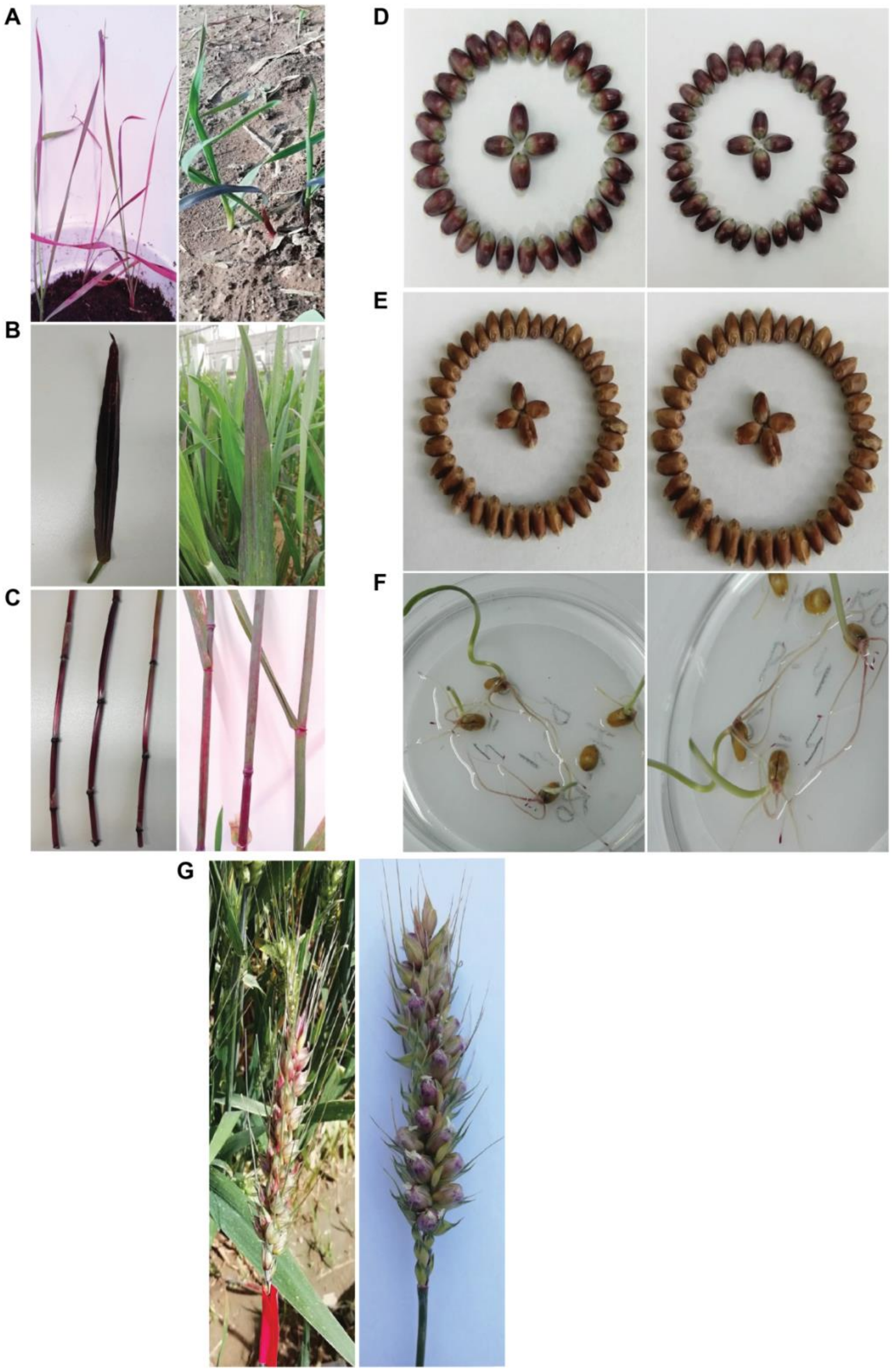

Figure S4. Anthocyanin production in the tissues of transgenic line AL-44. **A**: seedlings; **B**: leaves; **C**: stems; **D**: immature seeds; **E**: mature seeds; **F**: germinated roots; **G**: spikes.

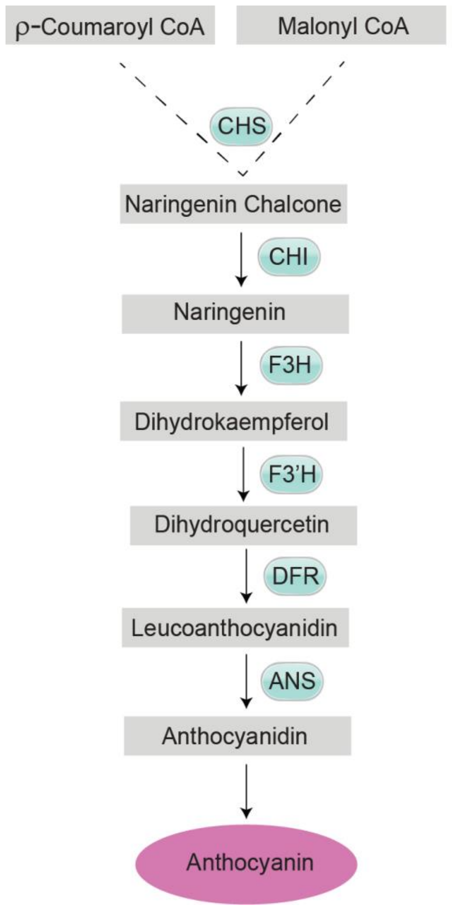

Figure S5. Structural genes involved in anthocyanin biosynthesis pathway in wheat.

A

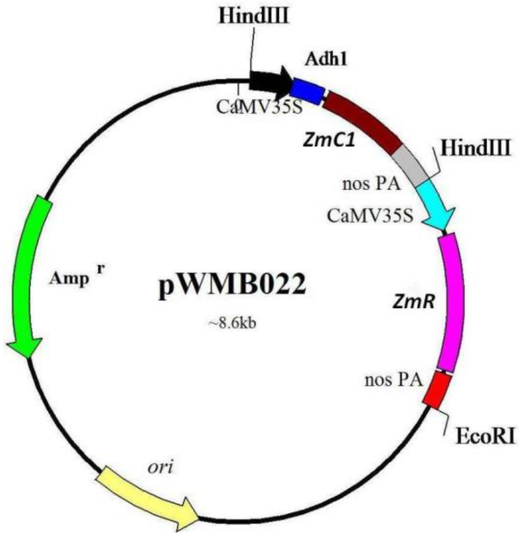

B

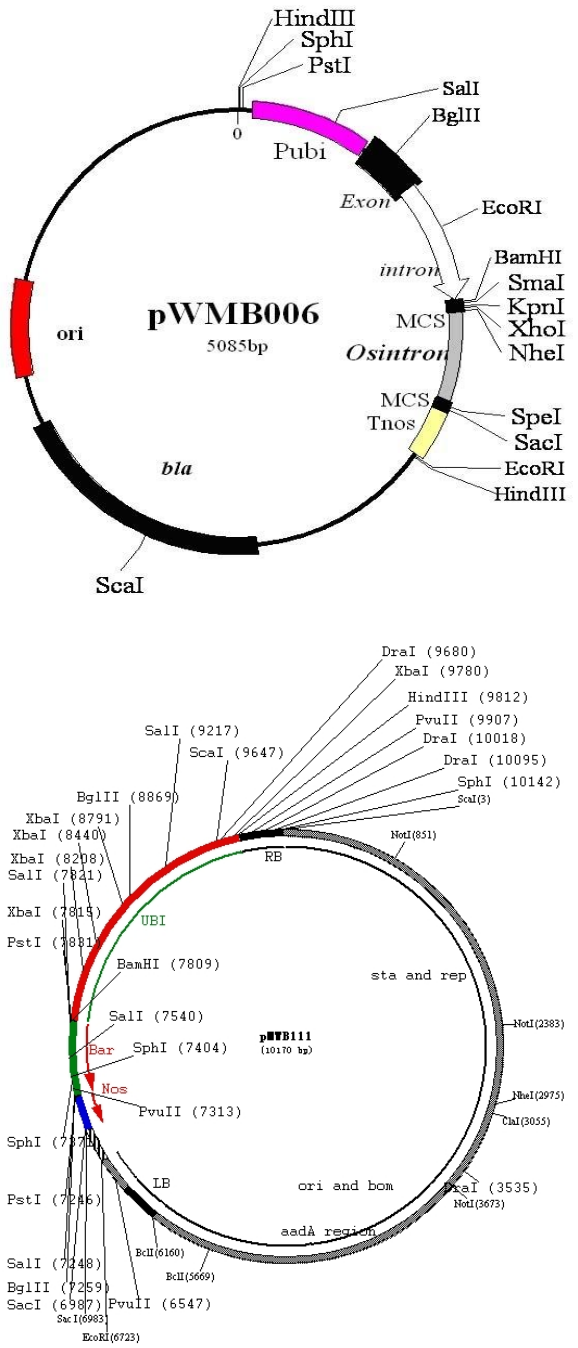

Figure S6. Plasmids pWMB022, pWMB006, and pWMB111 used in this study for constructing the expression vectors containing *ZmC1* and *ZmR* genes which are involved in anthocyanin biosynthesis pathway for wheat transformation. **A**: pWMB022; **B**: pWMB006; C: pWMB111 carrying *Bar* gene expression cassette.

Table S1. Determination of the anthocyanin contents in the transgene wheat lines.

Transgenic lines	Concentration anthocyanin contents (μg/g)	
	**Seeds**	**Leaves**
AL-44	10.177±0.180^{b}	96.517±3.328^{b}
AL-45	12.954±0.840^{a}	102.2±8.029^{a}
AL-30	0.0206±0.009^{c}	0.0307±0.005^{c}
AL-31	0.2283±0.0237^{c}	0.0678±0.036^{c}
AL-31	0.1541±0.023^{c}	0.0159±0.006^{c}
AL-40	0.054±0.070^{c}	0.8205±0.082^{c}
AL-41	0.2385±0.012^{c}	0.5608±0.041^{c}
Fielder	0.0861±0.032^{c}	0.1465±0.016^{c}
Jingdong18(JD18)	0.0243±0.008^{c}	0.0579±0.026^{c}

$^{a, b, c}$ Different letters indicate statistically significant differences ($P<0.05$).

Table 1. Primers used in this study for PCR and quantitative real-time PCR analysis.

Name	Forward (5′-3′)	Reverse (5′-3′)
ZmC1	GAAGGCGTTAAGAGAGGGGC	CCTGCTTCTCCTCCTCCTCCT
ZmR	CGACATAGAGGCGATGAC	ACTTGAGGACGAGGAACA
TaADP-RT	GCTCTCCAACAACATTGCCAAC	GCTTCTGCCTGTCACATACGC
ZmC1-RT	GTGGACGAGCAAGGAGGACGAT	CCTGTGGAGGCGGATGATGAGA
ZmR-RT	GGTTCTACAACGGCGAGGTGAA	CGGAAGGCGTAGGTCATGGAGA
TaC1-RT	GGTTGAGAGAGGGAGAGGGGAT	CGCAGACCAGCTCGCTGC
TaR-RT	CGGCCTGAGCAGTGTCATCA	GTGTCACGCTTTCAACAACATCC
TaCHS-RT	TGGAAAACAACCTACTACATACAGC	CAGACTAACAGGAGATCGAATGCAT
TaCHI-RT	CGTCCTCGTATTTGTCCGCTG	GAACCATAGTCACATATCACGAGG
TaF3H-RT	TGATTGATGCGTGGTGGGA	CAGAAACCAGTACGAAATATACGC
TaF3′H-RT	TAGCTCTCCAGTCTTCCTTGCC	GCCGTCGGCGTACACGA
TaDFR-RT	GCTCATCACAGGGAATGAAGC	GTCGTCGACGCCTGCGA
TaANS-RT	GAGGACGACCTGCTGCTGAA	GCCGTTGGTGAGGATGAAGGA

**RT*: Used for quantitative real-time PCR.
